# Irisin in Cartilage Homeostasis: Molecular Mechanisms and Therapeutic Implications for Osteoarthritis

**DOI:** 10.3390/jfmk11030343

**Published:** 2026-08-31

**Authors:** Raffaella Rosy Vescio, Morena Francesca Fiordalisi, Luca Ambrosio, Daniele Fiorentino, Elisabetta de Rinaldis, Giuseppina Di Giacomo, Fabrizio Russo, Vincenzo Denaro, Gianluca Vadalà, Rocco Papalia

**Affiliations:** 1 Laboratory for Regenerative Orthopaedics, Operative Research Unit of Orthopaedic and Trauma Surgery, Fondazione Policlinico Universitario Campus Bio-Medico, 00128 Rome, Italy; raffaella.vescio@policlinicocampus.it (R.R.V.); m.fiordalisi@policlinicocampus.it (M.F.F.); l.ambrosio@policlinicocampus.it (L.A.); g.digiacomo@unicampus.it (G.D.G.); fabrizio.russo@policlinicocampus.it (F.R.); r.papalia@policlinicocampus.it (R.P.); 2 Operative Research Unit of Laboratory, Fondazione Policlinico Universitario Campus Bio-Medico, 00128 Rome, Italy; 3 Research Unit of Orthopaedic and Trauma Surgery, Departmental Faculty of Medicine and Surgery, Università Campus Bio-Medico di Roma, 00128 Rome, Italy; danfior02@gmail.com (D.F.); derinaldiselisabetta@gmail.com (E.d.R.)

**Keywords:** irisin, osteoarthritis, chondrocytes, cartilage, myokine, extracellular matrix, exercise, autophagy, mitochondrial dysfunction, inflammation

## Abstract

Osteoarthritis (OA) is a leading cause of disability worldwide, characterized by progressive joint degeneration driven by extracellular matrix (ECM) breakdown, chondrocyte apoptosis, and chronic low-grade inflammation. Despite the availability of several pharmacological and non-pharmacological interventions, no current therapy effectively halts or reverses disease progression. Physical activity is traditionally recognized as a cornerstone in OA management, improving pain and functional outcomes; however, the molecular mechanisms underlying its beneficial effects remain partially understood. Irisin, a myokine generated by proteolytic cleavage of fibronectin type III domain-containing protein 5 in response to muscle contraction, has recently emerged as a potential regulator of cartilage homeostasis. Experimental evidence indicates that irisin exerts pleiotropic effects by modulating inflammatory and catabolic signaling pathways, promoting anabolic and reparative processes, restoring autophagic and mitophagic flux, and preserving mitochondrial function. These actions collectively contribute to the maintenance of ECM integrity and chondrocyte viability. In vitro and in vivo studies consistently support the protective role of irisin in cartilage biology, highlighting its involvement in the muscle–cartilage axis and its potential as both a biomarker and therapeutic target for OA. This narrative review critically evaluates the chondrocyte-specific mechanisms, receptor uncertainties, experimental-model limitations, analytical challenges, and delivery strategies that currently define the biological and translational relevance of irisin in OA. Particular attention is given to the identification of irisin-mediated signaling pathways in chondrocytes, optimization of delivery strategies, and the definition of exercise regimens capable of maximizing its chondroprotective effects.

## 1. Introduction

Osteoarthritis (OA) represents the most prevalent joint disorder and a leading cause of chronic pain and disability worldwide. Its global burden is continuously increasing, driven by population aging and the rising prevalence of metabolic disorders. In addition to its clinical impact, OA imposes a substantial socio-economic burden, with healthcare-related costs estimated at approximately $460 billion in 2019. It is estimated that more than 500 million people are affected globally, and these numbers are expected to further increase by 2050 [1]. Among the different anatomical sites, the knee is the most commonly affected joint, with radiographic and symptomatic OA reported in over 22% of individuals aged 40 years and older [2].

Traditionally regarded as a degenerative condition driven primarily by mechanical wear, OA is now increasingly recognized as a complex and multifactorial disease affecting the entire joint. It is characterized by progressive articular cartilage degradation, subchondral bone remodeling, synovial inflammation, osteophyte formation, and structural alterations in periarticular tissues. These pathological changes arise from interacting biomechanical, metabolic, and inflammatory mechanisms that progressively disrupt joint homeostasis. Despite its high prevalence, current therapeutic strategies remain largely symptomatic, focusing on pain relief and functional improvement rather than disease modification, before ultimately resorting to joint replacement at advanced stages. In this context, physical activity is consistently associated with improvements in pain, mobility, and overall joint function, and is therefore considered an important non-pharmacological component of OA management [3,4]. However, the molecular mediators linking mechanical stimulation to cartilage protection remain only partially understood.

The skeletal muscle has recently emerged as an active endocrine organ capable of secreting a wide range of bioactive molecules, namely myokines, in response to contraction. These muscle-derived factors contribute to inter-organ communication with adipose tissue, bone, and cartilage and include cytokines, growth factors, and myokines such as interleukin (IL)-6, myostatin, myonectin, and irisin [5,6,7,8]. Experimental evidence has also identified the skeletal muscle as a potential source of sclerostin, a secreted antagonist of Wnt/β-catenin signaling traditionally associated with osteocytes, supporting its proposed classification as a putative myokine involved in muscle-to-bone communication [9].

Irisin is a circulating peptide derived from fibronectin type III domain-containing protein 5 (FNDC5) cleavage, whose expression is regulated by peroxisome proliferator-activated receptor gamma coactivator-1 alpha (PGC-1α) during physical activity. Initially described for its role in energy metabolism and adipose tissue browning, irisin has subsequently been implicated in bone remodeling, muscle adaptation, and, more recently, cartilage biology [10,11]. Emerging evidence suggests that it modulates key processes involved in OA pathogenesis, including inflammation, extracellular matrix (ECM) turnover, mitochondrial function, and autophagy [12,13,14,15]. These findings suggest the existence of a muscle-cartilage axis, in which irisin acts as a molecular mediator linking physical activity to joint homeostasis. Nevertheless, several aspects remain unresolved, including the identification of its receptors in chondrocytes, the characterization of downstream signaling pathways, and its clinical relevance as a biomarker or therapeutic agent.

The aim of this review is to provide a comprehensive overview of the role of irisin in cartilage homeostasis and OA. We first summarize the main pathological and molecular features of OA, then discuss irisin biology and its systemic effects, and finally analyze the available experimental evidence supporting its chondroprotective role and therapeutic potential.

### Literature Search Strategy

This narrative review was conducted following a structured search of PubMed/MEDLINE, Scopus, and Web of Science from inception to 23 August 2026. The search strategy combined the key terms “irisin”, “FNDC5”, “osteoarthritis”, “cartilage”, “chondrocyte” with terms related to extracellular matrix, inflammation, autophagy, mitochondrial dysfunction, mechanical loading, integrins, and intra-articular delivery. Reference lists of relevant articles were manually screened to identify additional eligible studies. Priority was given to peer-reviewed original studies investigating the effects of irisin on chondrocytes, cartilage homeostasis, and experimental OA. Additional studies were included when relevant to irisin receptor biology, analytical measurement, exercise-mediated responses, or biomaterial-assisted delivery. Given the narrative design of the review, the available evidence was synthesized qualitatively, without conducting a formal risk-of-bias assessment or quantitative meta-analysis.

## 2. OA: Pathophysiological Background

OA is a chronic, progressive, and multifactorial disease that involves the entire joint. It is characterized by progressive degeneration of the articular cartilage, subchondral bone remodeling, aberrant angiogenesis, and synovial tissue activation. These pathological changes result in narrowing joint space, pain, stiffness, instability, and functional impairment, ultimately leading to reduced mobility and decreased quality of life [16,17,18].

### 2.1. Risk Factors

**Figure 1:**
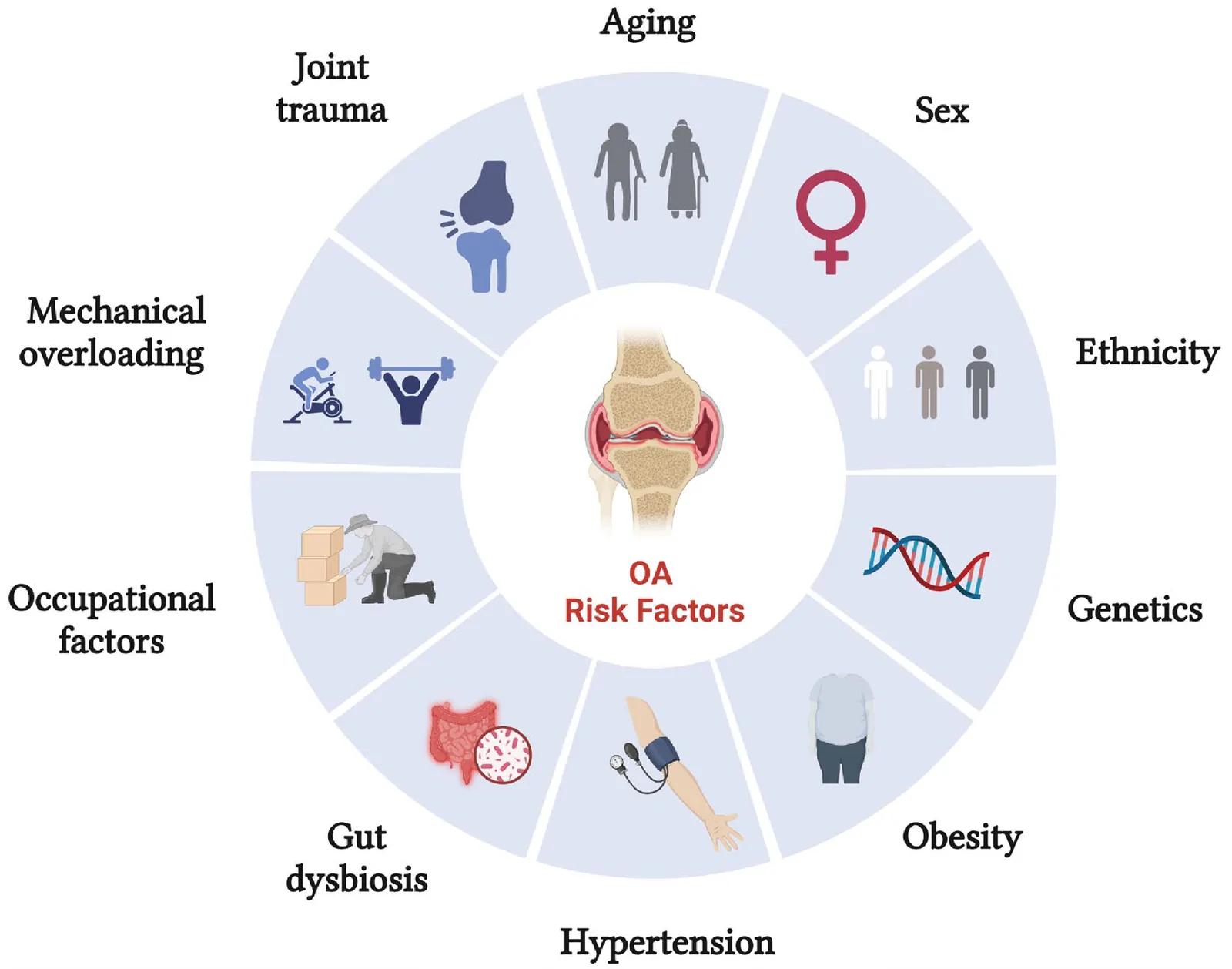
Overview of major risk factors associated with osteoarthritis (OA), including non-modifiable factors (age, sex, genetics, ethnicity) and modifiable factors (obesity, hypertension, gut microbiome, occupational loading, joint trauma, and physical activity).

The development of OA is influenced by a complex interplay of non-modifiable and modifiable risk factors that contribute to joint degeneration through mechanical, biological, and metabolic mechanisms (Figure 1) [17,19].

Non-modifiable factors include aging, female sex, genetic predisposition, and ethnicity. Aging represents the strongest risk factor and is associated with progressive chondrocyte senescence, reduced anabolic activity, impaired autophagy, and accumulation of oxidative damage, ultimately leading to ECM degradation and loss of tissue homeostasis [20]. Ethnicity has also been shown to influence OA prevalence, distribution, and severity, with significant variations observed across different populations, likely reflecting a combination of genetic susceptibility, lifestyle factors, and socio-environmental determinants [3,16,19]. Female sex, particularly in the post-menopausal period, is associated with an increased prevalence and severity of OA, largely attributable to estrogen deficiency [21].

Estrogens regulate joint homeostasis via estrogen receptors (ERα/ERβ) expressed in chondrocytes and subchondral bone cells, promoting ECM synthesis via production of type II collagen (COL2A1) and aggrecan (ACAN), while inhibiting catabolic enzymes such as matrix metalloproteinases (MMPs) and a disintegrin and metalloproteinase with thrombospondin motifs (ADAMTS). Following menopause, reduced estrogen levels lead to enhanced nuclear factor kappa B (NF-κB)-mediated inflammation, increased production of cytokines, including IL-1β, tumor necrosis factor (TNF)-α and IL-6, and altered bone remodeling, ultimately promoting cartilage degradation and chondrocyte apoptosis [22].

Genetic susceptibility also contributes significantly to OA risk, with heritability estimates of 40–65%. Variants in genes involved in ECM structure (e.g., COL2A1, ACAN), cartilage development (growth differentiation factor-5 [GDF5]), and signaling pathways (e.g., Wnt/β-catenin, SMAD3) have been associated with altered chondrocyte function, hypertrophy, and matrix turnover [23]. Additionally, polymorphisms in inflammatory genes further contribute to a pro-inflammatory joint environment, influencing disease onset and progression [24].

Among modifiable factors, obesity contributes to OA through both increased joint loading and systemic metabolic inflammation [25]. Adipose-derived mediators, including leptin, adiponectin, resistin, and visfatin, can influence chondrocyte metabolism, synovial inflammation, and subchondral bone remodeling [17,26,27,28]. Gut dysbiosis has also been proposed as a potential contributor through impaired intestinal barrier function, microbial-product translocation, and activation of systemic and local innate immune responses. However, the causal and clinical relevance of the gut–joint axis remains to be established [29,30,31].

Mechanical factors are central drivers of OA initiation and progression. Joint trauma, including ligamentous injury, meniscal damage and intra-articular fractures, disrupts joint stability and physiological load distribution, thereby triggering progressive degeneration of cartilage and subchondral bone [32]. Chronic excessive mechanical loading associated with occupational exposure or high-impact sports further accelerates tissue damage, whereas moderate physical activity appears to preserve joint function through maintenance of muscle strength and biomechanical homeostasis [33]. These factors collectively contribute to a complex and heterogeneous disease phenotype, highlighting the need for integrative therapeutic approaches.

### 2.2. OA Joint Changes and Pathophysiology

OA progression is characterized by coordinated structural, cellular, and biochemical alterations involving all joint tissues, including the articular cartilage, subchondral bone, and synovium. These changes reflect the breakdown of joint homeostasis and disruption of tightly regulated intercellular interactions.

The articular cartilage is a specialized, avascular, and aneural tissue composed of a dense ECM and a sparse population of chondrocytes. Under physiological conditions, chondrocytes maintain a dynamic balance between anabolic and catabolic processes, preserving ECM integrity through the synthesis of key structural components such as COL2A1 and ACAN, which confer tensile strength and resistance to compressive forces [34,35]. In early OA, cartilage undergoes subtle but critical biochemical changes, including proteoglycan depletion, increased tissue hydration, and disruption of the collagen network. Chondrocytes respond to mechanical and inflammatory stimuli by adopting a transient reparative phenotype; however, this response is often insufficient to restore tissue homeostasis [36]. As the disease progresses, chondrocytes acquire a hypertrophic and catabolic phenotype characterized by increased expression of matrix-degrading enzymes, including MMPs and aggrecanases (ADAMTS-4 and -5), along with elevated production of pro-inflammatory mediators. This shift results in progressive ECM breakdown, loss of cartilage elasticity, and structural weakening. In advanced stages, cartilage fibrillation, erosion, and full-thickness defects occur, ultimately leading to subchondral bone exposure [16,37].

The subchondral bone plays a critical role in load distribution and joint biomechanics and undergoes profound remodeling during OA progression. Early alterations include increased bone turnover, decreased bone mineral density, and microcrack formation, which may precede cartilage damage and alter load distribution. In later stages, the subchondral bone becomes progressively sclerotic, with increased mineralization, trabecular thickening, and reduced elasticity. This results in impaired shock absorption and further amplifies mechanical stress on the overlying cartilage [38]. In addition, osteophyte formation at joint margins represents a hallmark of OA and reflects aberrant endochondral ossification processes [16,36]. The bidirectional interaction between cartilage and subchondral bone, commonly referred to as cartilage–bone crosstalk, is increasingly recognized as a key driver of OA progression, although the underlying mechanisms remain incompletely understood [39].

The synovium contributes to OA from its early stages through synoviocyte hyperplasia, vascular remodeling, and immune-cell infiltration [40,41].

As OA advances, macrophage-enriched synovitis promotes the release of pro-inflammatory cytokines and chemokines, including IL-1β, TNF-α, and IL-6, which enhance chondrocyte catabolism and matrix-degrading enzyme production [42]. Synovial inflammation is therefore associated with both structural progression and pain severity [43,44]. Local biomechanical and metabolic factors further disrupt joint homeostasis [32]: malalignment, muscle weakness, and periarticular tissue dysfunction impair load distribution, whereas the infrapatellar fat pad releases adipokines and inflammatory mediators that reinforce synovial activation and cartilage degradation [45,46].

### 2.3. Molecular Mechanisms Involved in OA

OA progression is driven by a complex network of molecular pathways regulating inflammation, cellular metabolism, mechanotransduction, and ECM turnover. Rather than representing an isolated cartilage disorder, OA reflects the progressive breakdown of molecular homeostasis across the whole joint, in which chondrocytes, synoviocytes, subchondral bone cells, and immune mediators interact in a self-sustaining degenerative cascade.

#### 2.3.1. Inflammatory Signaling

Chronic low-grade inflammation is a central component of OA pathophysiology. NF-κB plays a dominant role. Its activation in chondrocytes and synovial cells induces the transcription of pro-inflammatory cytokines, including IL-1β, TNF-α, and IL-6, as well as catabolic enzymes such as MMP-1, MMP-3, MMP-13 and aggrecanases (ADAMTS-4 and ADAMTS-5) [47,48]. These mediators promote ECM degradation, suppress anabolic gene expression, and contribute to chondrocyte apoptosis and senescence [49]. In addition, they contribute to chondrocyte dysfunction by promoting cellular senescence and apoptosis. Inflammatory cytokines, together with reactive oxygen species (ROS) and nitric oxide (NO), act as potent inducers of apoptotic pathways in OA cartilage, modulating the balance between pro-apoptotic factors (e.g., BAX) and anti-apoptotic proteins (e.g., BCL-2), and ultimately activating caspase-dependent mechanisms leading to chondrocyte death [50].

Mitogen-activated protein kinase (MAPK) signaling, particularly through p38, c-Jun N-terminal kinase (JNK), and extracellular signal-regulated kinase (ERK), is also critically involved in OA. These pathways are activated by inflammatory cytokines, oxidative stress, and mechanical injury, and amplify the catabolic response by enhancing expression of MMPs, ADAMTS, and inflammatory mediators. Together, NF-κB and MAPK signaling constitute a major inflammatory axis driving cartilage degeneration [22,47].

#### 2.3.2. ECM Degradation and Chondrocyte Phenotypic Shift

A hallmark of OA is the imbalance between ECM synthesis and degradation. In response to mechanical and inflammatory stress, chondrocytes undergo a phenotypic shift from a quiescent homeostatic state toward a hypertrophic and catabolic phenotype. This phenotypic transition is characterized by reduced synthesis of COL2 and ACAN, together with increased production of matrix-degrading enzymes and hypertrophic markers such as type X collagen (COL10) [51]. This process is closely linked to dysregulation of signaling pathways involved in cartilage differentiation and maintenance. The Wnt/β-catenin pathway, which is essential for skeletal development and cartilage homeostasis, becomes aberrantly activated in OA. Excessive Wnt/β-catenin signaling promotes chondrocyte hypertrophy, terminal differentiation, and expression of catabolic mediators, thereby contributing to cartilage loss and osteophyte formation [52,53].

#### 2.3.3. Autophagy, Cellular Senescence, and Metabolic Stress

Impaired cellular quality-control mechanisms are increasingly recognized as key contributors to OA progression. Autophagy, a lysosome-dependent process responsible for the removal of damaged proteins and organelles, is essential for chondrocyte survival under stress. In OA, autophagic activity is progressively reduced, leading to accumulation of cellular damage, mitochondrial dysfunction, and increased susceptibility to apoptosis. [54]. The mechanistic target of rapamycin (mTOR)/adenosine monophosphate-activated protein kinase (AMPK) signaling axis is a central regulator of these processes. mTOR overactivation suppresses autophagy and promotes cellular senescence, matrix degradation, and metabolic dysfunction. In contrast, AMPK exerts protective effects by promoting autophagy, improving energy homeostasis, and limiting oxidative stress. Decreased AMPK activity has been consistently associated with OA, suggesting that disruption of the mTOR/AMPK balance contributes to disease progression [44,55,56].

Chondrocyte senescence further amplifies this process. Senescent chondrocytes exhibit reduced proliferative and anabolic capacity while maintaining high production of inflammatory cytokines and matrix-degrading enzymes, a phenotype consistent with the senescence-associated secretory phenotype (SASP). This senescent state promotes ECM breakdown and perpetuates chronic joint inflammation [57].

#### 2.3.4. Oxidative Stress and Mitochondrial Dysfunction

Oxidative stress is another major molecular driver of OA. Excessive generation of ROS, combined with impaired antioxidant defenses, results in oxidative damage to proteins, lipids, mitochondrial components, and DNA. In chondrocytes, increased ROS levels impair anabolic responses, promote inflammatory signaling, and activate apoptosis-related pathways [17,56].

Mitochondrial dysfunction is tightly linked to oxidative stress in OA. Altered mitochondrial biogenesis, impaired membrane potential, reduced ATP production, and defective mitophagy contribute to loss of chondrocyte viability and metabolic competence. Since the articular cartilage is highly dependent on cellular stress adaptation for long-term maintenance, mitochondrial failure plays a key role in accelerating tissue degeneration [56].

#### 2.3.5. Innate Immunity and Danger Signaling

Innate immune pathways contribute to OA by linking tissue damage to persistent inflammation. Damage-associated molecular patterns (DAMPs), released from degraded ECM or stressed cells, activate pattern-recognition receptors such as toll-like receptors (TLRs) on chondrocytes, synoviocytes, and immune cells. This stimulation amplifies NF-κB–dependent inflammatory signaling and promotes the production of cytokines, chemokines, and proteolytic enzymes [58]. In parallel, microbial products such as LPS, potentially derived from gut dysbiosis, may further enhance systemic and local inflammation, supporting the involvement of the gut–joint axis in OA progression [31]. Collectively, these mechanisms result in the amplification of the inflammatory microenvironment within the joint, characterized by increased recruitment and activation of innate immune cells, including macrophages and neutrophils, and enhanced production of pro-inflammatory mediators such as IL-1β, TNF-α, and IL-6. This sustained inflammatory state promotes synovial activation, exacerbates chondrocyte catabolic responses, and accelerates ECM degradation, thereby contributing to progressive joint damage and symptom severity [59].

#### 2.3.6. Synovitis and Inter-Tissue Crosstalk

Synovial inflammation is not merely a secondary reaction to cartilage damage, but an active component of disease progression. Activated synovial fibroblasts and infiltrating macrophages release cytokines, chemokines, prostaglandins, and proteases that exacerbate cartilage catabolism and contribute to pain sensitization. This inflammatory synovial microenvironment also influences subchondral bone remodeling and may enhance vascular invasion and osteophyte formation [60,61]. At the same time, increasing evidence supports the existence of reciprocal crosstalk among cartilage, subchondral bone, synovium, and periarticular tissues. These interactions are mediated by soluble factors, extracellular vesicles, and biomechanical signals, further reinforcing the concept of OA as a whole-joint disease [36].

### 2.4. Translational Relevance

Collectively, these molecular pathways indicate that OA progression arises from the combined effects of inflammatory signaling, metabolic dysregulation, oxidative stress, impaired autophagy, and altered mechanotransduction. This multifactorial complexity partly explains why current therapeutic approaches remain largely symptomatic and fail to effectively halt or reverse disease progression [62].

This therapeutic gap highlights the need for disease-modifying strategies capable of targeting multiple interconnected pathways simultaneously. In this regard, increasing attention has shifted toward endogenous regulators of tissue homeostasis that operate at the interface between systemic metabolism and local joint biology. The skeletal muscle has emerged as a key endocrine organ involved in inter-organ communication through the secretion of myokines released during physical activity. These factors exert autocrine, paracrine, and endocrine effects, enabling skeletal muscle to modulate distant tissues, including the adipose tissue, liver, bone, and joints, and to orchestrate adaptive responses to physical activity [6].

Among these mediators, irisin has attracted particular interest as a potential molecular link between skeletal muscle activity and joint health [63]. By concurrently modulating key processes involved in OA pathophysiology, such as inflammation, mitochondrial function, autophagy, and ECM turnover, irisin emerges as a biologically plausible integrator of the protective effects of physical activity on cartilage. As such, it represents a promising candidate for the development of novel disease-modifying approaches in OA.

## 3. The Role of Irisin in Musculoskeletal Health

**Figure 2:**
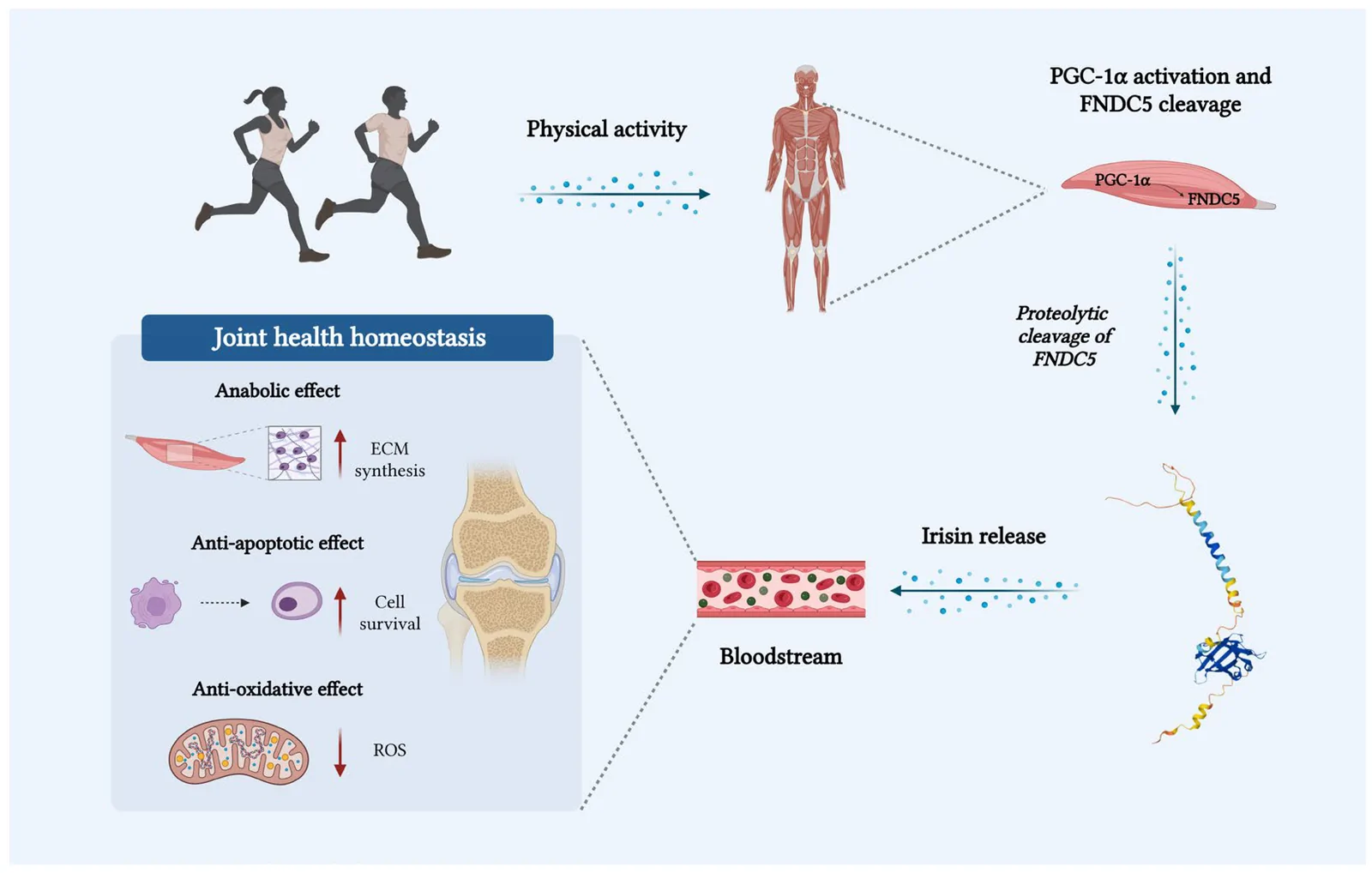
Schematic representation of the muscle–cartilage axis activated by physical activity. Exercise stimulates peroxisome proliferator-activated receptor gamma coactivator-1 alpha (PGC-1α), promoting fibronectin type III domain-containing protein 5 (FNDC5) expression and its proteolytic cleavage into irisin. Circulating irisin is released into the bloodstream and exerts systemic effects on joint tissues. At the cartilage level, irisin contributes to joint homeostasis by enhancing extracellular matrix (ECM) synthesis (anabolic effect), promoting chondrocyte survival (anti-apoptotic effect), and reducing oxidative stress through decreased reactive oxygen species (ROS) production.

Irisin, first identified in 2012 [10], is a peptide hormone predominantly secreted by the skeletal muscle in response to physical activity and characterized by pleiotropic systemic effects on metabolism and musculoskeletal homeostasis [7,8,11]. It originates from the transmembrane precursor FNDC5, a 209–212 amino acid protein with a fibronectin III structural domain. Proteolytic cleavage of the extracellular portion of FNDC5 generates irisin, a circulating peptide of 112 amino acids with two glycosylation sites [10,15]. Once released into the bloodstream, irisin acts as a hormone-like mediator linking muscle activity to systemic adaptations [64,65]. Although the skeletal muscle represents its primary source, irisin expression has also been detected in adipose tissue, bone, brain, and liver, supporting its role in inter-organ communication and metabolic regulation (Figure 2) [66,67].

Exercise promotes irisin release through FNDC5 cleavage, a process largely regulated by PGC-1α [66]. Activated during muscle contraction, PGC-1α enhances FNDC5 transcription, often in cooperation with the transcription factor cAMP response element-binding protein (CREB), leading to increased circulating myokine levels [11]. Although FNDC5 expression is commonly considered a marker of irisin production, circulating irisin levels are also influenced by post-translational processing and secretion dynamics [12]. Overall, activation of the PGC-1α–FNDC5 axis represents the main mechanism linking physical activity to systemic irisin signaling and its potential musculoskeletal effects [10].

Irisin has attracted considerable attention for its pleiotropic actions across multiple tissues [17]. In adipocytes, it enhances lipolysis through activation of the cyclic adenosine monophosphate (cAMP)–protein kinase A (PKA)–hormone-sensitive lipase (HSL) signaling pathway [12]. Increased intracellular cAMP levels activate PKA, which phosphorylates HSL, leading to triglyceride hydrolysis into free fatty acids and glycerol. In parallel, irisin induces browning of white adipose tissue by upregulating uncoupling protein 1 (UCP1), thereby enhancing thermogenesis and energy expenditure [16]. It also attenuates lipid accumulation and oxidative stress through modulation of metabolic regulators such as protein arginine methyltransferase 3 (PRMT3) [11].

In the skeletal muscle, irisin enhances oxidative metabolism, promotes mitochondrial biogenesis, and contributes to adaptive responses to exercise, including muscle hypertrophy [13,17]. In the bone, irisin exerts consistent anabolic effects by promoting osteoblast proliferation, differentiation, and matrix mineralization, thereby sustaining bone remodeling and skeletal homeostasis [64,68]. It enhances bone mineral density and mechanical strength by stimulating osteoblast activity, limiting osteoclast-mediated resorption, and improving subchondral bone microarchitecture [69]. Mechanistically, irisin activates multiple signaling pathways, including ERK1/2, activating transcription factor 4 (ATF4), p38 MAPK, AMPK, and Wnt/β-catenin signaling, which collectively promote osteogenesis [65,70]. In parallel, it inhibits osteoclastogenesis through suppression of the receptor activator of nuclear factor kappa B ligand (RANKL)/nuclear factor of activated T cells cytoplasmic 1 (NFATc1) pathway and reduces oxidative stress–mediated signaling [69]. Additional effects include attenuation of osteoblast senescence via downregulation of cyclin-dependent kinase inhibitor p21 and modulation of cellular metabolism through nuclear factor erythroid 2-related factor 2 (Nrf2) activation [71,72]. Beyond the bone, irisin has been shown to play a direct role in cartilage biology. It regulates chondrocyte proliferation and apoptosis, attenuates inflammation-induced catabolic responses, and preserves the ECM structural integrity. Irisin exerts protective effects on cartilage by reducing the production of inflammatory mediators, such as IL-1 and IL-6, preventing chondrocyte apoptosis and supporting cell proliferation [73]. It also downregulates matrix-degrading enzymes, including MMP-1, MMP-13, and ADAMTS-5 [74]. By downregulating these catabolic mediators, irisin reduces the breakdown of cartilage-specific ECM proteins, most notably COL2A1 and ACAN, while simultaneously supporting anabolic processes that sustain chondrocyte function and matrix synthesis [15,75,76]. At the molecular level, irisin modulates several interconnected pathways involved in cartilage degeneration and chondrocyte survival. It suppresses NF-κB signaling by inhibiting p65 nuclear translocation and attenuates MAPK activation (p38, JNK, and ERK), thereby reducing the expression of pro-inflammatory cytokines, catabolic mediators, and apoptosis-related genes [76]. Furthermore, it can inhibit nucleotide-binding oligomerization domain-like receptor family pyrin domain containing 3 (NLRP3) inflammasome activation, reducing pyroptotic cell death and modulates the AMPK/mTOR axis, enhancing autophagy and cell stress resistance. Collectively, these effects preserve chondrocyte viability and support ECM maintenance, contributing to cartilage integrity and joint function [16,37,77].

The biological effects of irisin on joint tissues also appear to depend on the magnitude and quality of mechanical loading. Physiological exercise promotes irisin release and supports cartilage homeostasis through anti-inflammatory and anabolic responses, including enhanced ECM synthesis. Conversely, excessive or abnormal mechanical loading may activate catabolic signaling pathways, such as aberrant Wnt/β-catenin activation, increase IL-1β–mediated inflammatory responses, and accelerate matrix degradation, thereby contributing to OA progression [78,79,80].

### 3.1. The Effect of Irisin on Chondrocytes

Irisin directly modulates chondrocyte function and plays a key role in maintaining cartilage homeostasis under both physiological and pathological conditions [55,81]. During OA progression, chondrocytes undergo phenotypic and metabolic alterations characterized by enhanced catabolic activity, ECM degradation, and impaired tissue repair capacity. Irisin appears to counterbalance these processes through coordinated regulation of inflammatory signaling, mitochondrial function, and extracellular matrix homeostasis.

At the mitochondrial level, irisin enhances cellular bioenergetics and redox balance by activating PGC-1α–mitochondrial transcription factor A (TFAM) signaling and modulating sirtuin 3 (SIRT3). It promotes mitochondrial fusion through upregulation of mitofusin 1 (MFN1) and limits excessive fission mediated by dynamin-related protein 1 (DRP1), thereby preserving mitochondrial integrity and ATP production. In parallel, irisin restores autophagic and mitophagic flux by regulating microtubule-associated protein 1 light chain 3 beta II (LC3-II), autophagy-related proteins (ATGs), and the PTEN-induced kinase 1 (PINK1)/Parkin pathway, ensuring efficient removal of damaged organelles and cellular stress adaptation [53,82].

Irisin also exerts potent anti-inflammatory and anti-catabolic effects in chondrocytes. It suppresses NF-κB activation by preventing p65 nuclear translocation and attenuates MAPK signaling, including JNK and p38 MAPK pathways [74,83]. Under IL-1β–stimulated conditions, irisin further inhibits the phosphoinositide 3-kinase/protein kinase B (PI3K/Akt)/NF-κB axis and limits activation of the NLRP3 inflammasome, thereby reducing inflammatory mediator release and pyroptotic cell death. In addition, irisin modulates signaling pathways involved in cartilage degeneration, including aberrant Wnt/β-catenin activation, contributing to the restoration of the balance between anabolic and catabolic activities in OA chondrocytes [74,76,84].

At the ECM level, irisin promotes a shift toward an anabolic phenotype by upregulating cartilage-specific genes, including COL2A1 and ACAN, as well as tissue inhibitors of metalloproteinases (TIMPs). Concurrently, it suppresses matrix-degrading enzymes such as MMP-1, MMP-13 and ADAMTS-5, along with hypertrophic markers including collagen type X alpha 1 chain (COL10A1). This coordinated regulation preserves ECM integrity and prevents cartilage breakdown [65].

**Figure 3:**
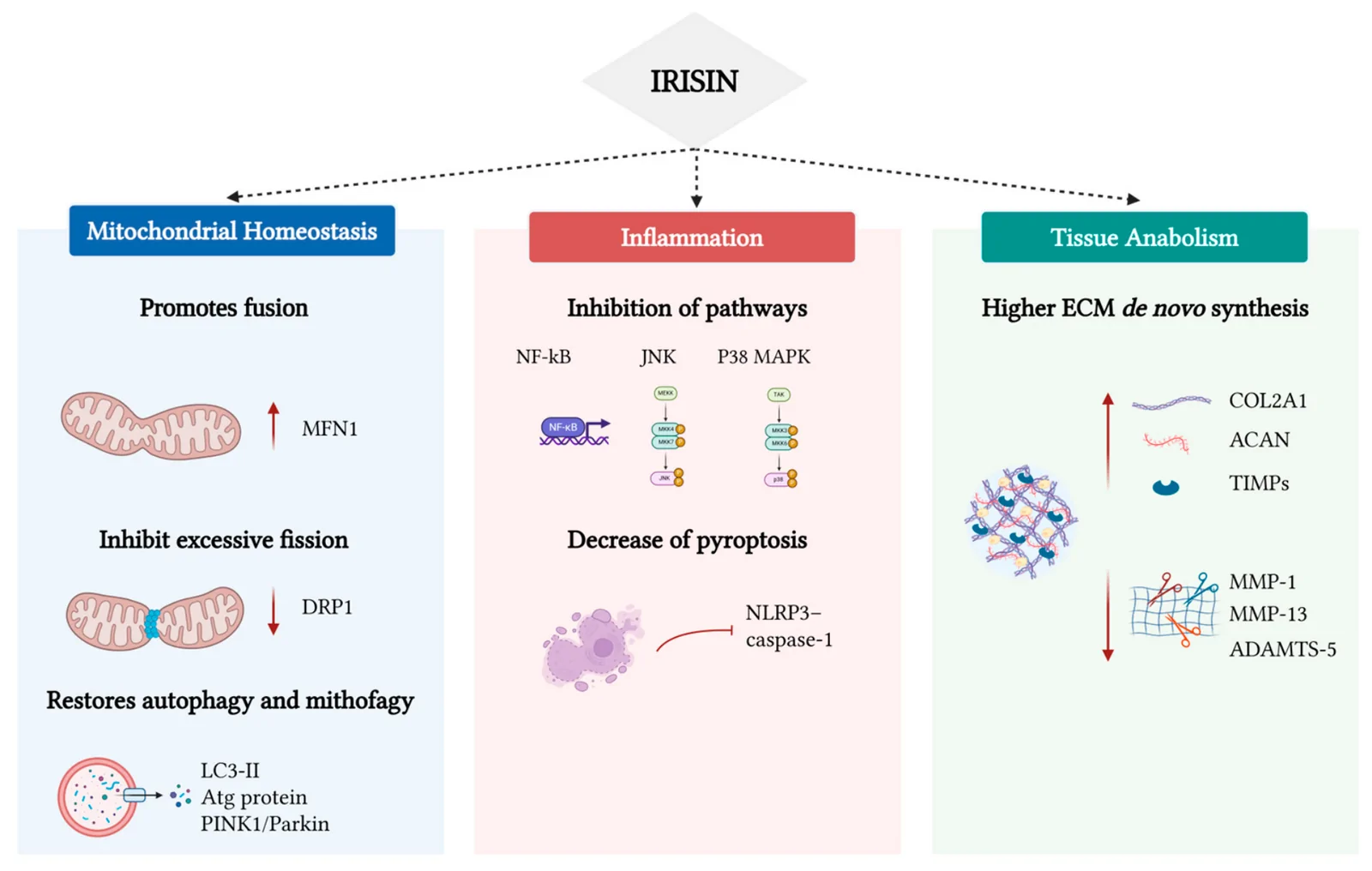
Molecular mechanisms of irisin-mediated chondroprotection. Irisin regulates cartilage homeostasis by enhancing mitochondrial function (↑ MFN1, ↓ DRP1), restoring autophagy and mitophagy (LC3-II, ATGs, PINK1/Parkin), inhibiting inflammatory signaling (NF-κB, JNK, p38 MAPK) and NLRP3 inflammasome activation, and promoting ECM anabolism (↑ COL2A1, ACAN, TIMPs; ↓ MMPs, ADAMTS-5). Abbreviations: ACAN: aggrecan; ADAMTS: a disintegrin and metalloproteinase with thrombospondin motifs; COL2A1: type II collagen alpha 1 chain; DRP1: dynamin-related protein 1; ECM: extracellular matrix; JNK: c-Jun N-terminal kinase; LC3-II: light chain 3 beta II; MFN1: mitofusin-1; MMPs, matrix metalloproteinases; NF-κB, nuclear factor kappa B; NLRP3: NOD-, LRR- and pyrin domain-containing protein 3; PINK1: PTEN-induced kinase 1; p38 MAPK, p38 mitogen-activated protein kinase; TIMPs, tissue inhibitors of metalloproteinases.

Collectively, irisin acts as a multifunctional regulator of chondrocyte homeostasis by integrating mitochondrial protection, autophagy regulation, anti-inflammatory signaling, and ECM remodeling (Figure 3). Through these mechanisms, it counteracts the key molecular drivers of OA progression and supports cartilage integrity. Although integrin complexes, particularly αVβ5 and αVβ1, have been proposed as candidate irisin receptors in other tissues, their role in chondrocyte signaling remains to be fully elucidated [85].

#### 3.1.1. Candidate Integrin Receptors and Mechanical Regulation

The receptor system responsible for irisin signaling in articular chondrocytes remains unresolved. In osteocytes, quantitative proteomic screening identified αV-containing integrins as candidate mediators of irisin signaling. Subsequent biochemical binding analyses demonstrated that recombinant irisin bound αVβ5 more strongly than the other integrin heterodimers examined, while pharmacological blockade of αV integrins attenuated selected irisin-induced signaling and cellular responses [86]. Evidence from mature human adipocytes further supports a receptor role for integrin αV [87], with αVβ5 currently representing the most extensively characterized integrin receptor for irisin [88].

Human articular chondrocytes express several αV-containing heterodimers, including αVβ1, αVβ3, and αVβ5, with αV-containing integrins being particularly represented in the superficial cartilage zone [88]. Recombinant irisin also induces rapid ERK1/2 phosphorylation and chondrogenic responses in three-dimensional human chondrocyte cultures [89]. Based on observations in other cell types, irisin binding to αVβ5 could promote a signaling-competent integrin conformation and receptor clustering at the plasma membrane, followed by recruitment of focal-adhesion-associated proteins and activation of FAK/Src–ERK signaling [86,90].

The composition and mechanical state of the ECM may influence this putative receptor system. αV-containing integrins mediate interactions with RGD-containing proteins, including fibronectin, vitronectin, and osteopontin [90]. OA-associated matrix remodeling may therefore modify integrin ligand occupancy, conformational state, clustering, and membrane organization, thereby altering the cellular context in which irisin signaling occurs.

Mechanical loading may further regulate the integrin environment through acute changes in receptor activation and longer-term modulation of integrin expression. Evidence from α5β1-mediated mechanotransduction demonstrates that cyclic mechanical strain activates focal-adhesion-associated and IL-4-dependent signaling in human articular chondrocytes, producing matrix-preserving responses that are impaired in OA cells [91,92,93]. A complementary role for αVβ5 has been identified in load-dependent ECM regulation. In normal human articular chondrocytes, mechanical stimulation enhanced glycosaminoglycan synthesis and newly synthesized aggrecan production, whereas functional blockade of αVβ5 abrogated these anabolic responses. Conversely, OA chondrocytes exhibited reduced basal glycosaminoglycan (GAG) production and failed to increase matrix synthesis following mechanical stimulation [94].

Mechanical loading also differentially regulates α5- and β1-integrin expression in cartilage explants, confirming that the integrin profile of chondrocytes is mechanically responsive [95]. Under excessive mechanical stress, αVβ3- and αVβ5-associated signaling has been linked to phosphorylation of FAK, ERK, JNK, and p38 and to increased expression of IL-1β, TNF-α, MMP-3, and MMP-13. Pharmacological inhibition with cilengitide attenuated these inflammatory and catabolic responses [96]. Because cilengitide inhibits both αVβ3 and αVβ5, this experiment demonstrates the involvement of αV-integrin-associated signaling without distinguishing the individual contribution of either heterodimer.

Additional evidence indicates that excessive loading can increase αVβ3 expression and FAK–ERK1/2 activation in chondrocytes, whereas pharmacological inhibition of αVβ3 attenuates hypertrophic and matrix-catabolic responses both in vitro and in vivo [97]. Mechanically induced changes in αVβ3 may therefore modify the relative abundance, organization, or downstream signaling of αV-containing heterodimers and influence the cellular environment in which irisin acts. This possibility should be interpreted as a hypothetical remodeling of the αV-integrin signaling landscape rather than as evidence of direct competition between αVβ3 and αVβ5.

Collectively, these findings support a model in which mechanical loading and OA-associated matrix remodeling influence chondrocyte sensitivity to irisin by modifying the abundance, activation state, ligand occupancy, and membrane organization of αV-containing integrins. However, direct irisin–αVβ5 binding and αVβ5-dependent target engagement have not been conclusively demonstrated in articular chondrocytes.

#### 3.1.2. In Vitro Studies

Available in vitro evidence indicates that irisin directly modulates chondrocyte function and may contribute to muscle-cartilage crosstalk. Across different cellular models, irisin has been associated with matrix-anabolic, anti-catabolic, anti-inflammatory, and cytoprotective responses. However, differences in cell source, culture system, recombinant protein preparation, concentration, and exposure duration require cautious comparison among studies.

Vadalà et al. [74] investigated for the first time the impact of irisin on human chondrocytes derived from OA patients, cultured in a 3D pellet system. Cells were treated either with phosphate-buffered saline (control) or human recombinant irisin at different concentrations and characterized at cellular, biochemical and molecular levels. Treatment with recombinant irisin at low concentrations (25 ng/mL) significantly increased glycosaminoglycan (GAG) production, indicating enhanced proteoglycan synthesis. Moreover, cell content was higher in irisin-treated pellets than in controls after 14 days of culture, suggesting preserved cell viability under the experimental conditions examined. At the molecular and protein level, irisin upregulated COL2 while suppressing COL10, thereby promoting a hyaline-like, non-hypertrophic phenotype. In parallel, it reduced the expression of pro-inflammatory cytokines (IL-1β, IL-6), catabolic enzymes (MMP-1, MMP-13), and iNOS, while increasing TIMP-1 and TIMP-3. These effects were associated with inhibition of MAPK and NF-κB signaling pathways, indicating a combined anabolic and anti-catabolic action.

Complementary findings were reported by Li et al. [84] using human SW1353 chondrosarcoma cells exposed to IL-1β to reproduce selected OA-like inflammatory responses. IL-1β reduced type II collagen and increased MMP-13 expression while activating Wnt/β-catenin and NF-κB signaling. Irisin partially reversed these alterations by restoring type II collagen, suppressing MMP-13, and reducing Wnt-1, β-catenin, and phosphorylated NF-κB p65. In a complementary experiment, cells were treated with lithium chloride (LiCl), a pharmacological activator of Wnt/β-catenin signaling. Irisin attenuated LiCl-induced pathway activation, further supporting its modulatory effect on this catabolic signaling cascade. However, the study presents an internal inconsistency in the reported irisin units: most experiments indicate concentrations in the millimolar range, whereas the LiCl experiment reports 20 nM. Given the apparent biological implausibility of millimolar irisin concentrations, the exposure conditions and physiological relevance of these findings should be interpreted cautiously.

More recently, Posa et al. [98] examined the effects of recombinant irisin on primary human articular chondrocytes from non-OA donors maintained in three-dimensional pellet cultures. Irisin exposure (100 ng/mL) increased SOX9, ACAN, and COL2A1 expression and enhanced COL2 deposition. Irisin-treated pellets also exhibited a greater cross-sectional area, potentially reflecting increased cell proliferation and/or ECM accumulation. However, neither COL10A1 expression nor the proportion of Safranin O-positive ECM differed significantly between treated and control pellets. At the signaling level, irisin induced rapid and transient ERK1/2 phosphorylation, peaking at 10 min and returning towards baseline by 20 min. Although these kinetics are consistent with receptor-initiated signal transduction, neither the molecular identity of the receptor nor its functional involvement in the observed chondrogenic response was established [98,99].

Further mechanistic insight was provided by Chen et al. [75] using human mesenchymal stromal cells (hMSCs). Irisin treatment enhanced chondrogenesis, as demonstrated by increased pellet size and upregulation of COL2A1, ACAN, and SOX9. Transcriptomic analysis revealed activation of the Ras-related protein 1 (Rap1)/PI3K/Akt pathway through suppression of signal-induced proliferation-associated 1 like 2 (SIPA1L2). Notably, irisin upregulated microRNA-125b-5p (miR-125b-5p), which directly targets SIPA1L2, thereby sustaining pathway activation and promoting chondrogenic differentiation. This identifies a novel miR-125b-5p–SIPA1L2–Rap1/PI3K/Akt axis as a key mediator of irisin-induced cartilage regeneration.

Additional evidence was provided by Jia et al. [76], who investigated primary rat articular chondrocytes pretreated with irisin at 5 or 10 ng/mL before stimulation with IL-1β. Irisin preserved type II collagen expression while reducing MMP-13 and ADAMTS-5. It also attenuated inflammatory responses and chondrocyte pyroptosis through inhibition of PI3K/Akt/NF-κB signaling and suppression of the NLRP3/caspase-1 pathway. These findings identify a potential role for irisin in modulating inflammasome-associated cell death, although their relevance to human OA chondrocytes remains to be established.

Similarly, Wang et al. [82] characterized the effects of irisin on mitochondrial homeostasis and autophagic flux in primary mouse articular chondrocytes challenged with IL-1β. Irisin attenuated oxidative stress, preserved mitochondrial membrane potential and ATP production, and partially reversed the IL-1β-mediated suppression of GAG synthesis and cartilage-associated marker expression. These responses were associated with recovery of SIRT3 and UCP1 expression, improved mitochondrial dynamics, enhanced autophagic and mitophagic activity, and reduced intracellular ROS accumulation. Complementary analyses of human OA cartilage demonstrated lower FNDC5 and LC3-II expression in structurally damaged regions; however, these specimens were analyzed observationally and were not exposed to irisin in vitro.

Collectively, these findings demonstrate that irisin exerts coordinated anabolic, anti-inflammatory, anti-catabolic, and cytoprotective effects on chondrocytes through multiple converging signaling pathways, including MAPK, NF-κB, Wnt/β-catenin, PI3K/Akt, inflammasome-related pathways, and mitochondrial-autophagy regulatory networks. These observations support the role of irisin as a key regulator of cartilage homeostasis and a promising therapeutic target in OA. A summary of the actual in vitro evidence on the effect of irisin on cartilage homeostasis is depicted in Table 1.

#### 3.1.3. In Vivo Studies

Experimental in vivo evidence has been obtained primarily from surgically induced murine models of OA. Destabilization of the medial meniscus (DMM) and anterior cruciate ligament transection (ACLT) reproduce selected features of slowly progressive and post-traumatic OA, respectively, although neither model fully reflects the chronic and heterogeneous nature of human disease [65].

Li et al. [81] examined the contribution of FNDC5/irisin signaling using irisin-knockout and irisin-knock-in mice, together with surgically induced OA and intra-articular recombinant irisin administration. Genetic deletion of irisin exacerbated cartilage degeneration, as indicated by higher Osteoarthritis Research Society International (OARSI) scores, reduced COL2A1 and ACAN expression, and increased expression of the hypertrophic marker COL10A1. Conversely, genetic irisin overexpression and repeated intra-articular administration of recombinant irisin attenuated cartilage erosion and promoted a more matrix-anabolic phenotype [65]. These findings support an association between enhanced FNDC5/irisin signaling and preservation of cartilage homeostasis. However, because treatment was initiated shortly after joint injury, the results indicate modulation of early experimental OA development rather than reversal of established disease.

Wang et al. evaluated intra-articular recombinant irisin in the murine DMM model [82]. A single injection administered one week after surgery reduced cartilage erosion, synovial inflammation, oxidative DNA damage, apoptosis, and histological OA severity, while partially improving gait parameters. These effects were accompanied by increased expression of microtubule-associated protein 1 light chain 3 beta II (LC3-II), sirtuin 3 (SIRT3), and uncoupling protein 1 (UCP1), consistent with enhanced autophagic activity and improved mitochondrial homeostasis. Complementary experiments in primary murine chondrocytes showed preservation of mitochondrial membrane potential and ATP production, reduction of reactive oxygen species, and normalization of mitochondrial dynamics and mitophagic flux. The source-reported intra-articular regimen consisted of recombinant irisin at 20 mg/mL in a 10-μL injection, corresponding to 200 μg per joint [82]. This concentration was retained as reported in the original study; however, the absence of dose-ranging and local pharmacokinetic analyses limits direct cross-study comparison.

Evidence for a reparative effect has also been reported in a rat full-thickness osteochondral-defect (FTOD) model [100]. Following bone-marrow stimulation, intra-articular irisin improved selected macroscopic, histological, and micro-computed tomography indices of osteochondral repair. Combined administration of irisin and bevacizumab produced the most favorable overall outcomes, including lower Pineda scores and reduced defect dimensions [100]. However, this model evaluates the repair of an acute focal osteochondral lesion rather than the treatment of chronic OA. Moreover, because a formal pharmacological interaction was not established, the results should not be interpreted as definitive evidence of synergy or regeneration of native hyaline cartilage.

Exercise-based models provide indirect evidence for the involvement of endogenous irisin in muscle–cartilage communication. In rats with monoiodoacetate (MIA)-induced knee OA, treadmill exercise and electrically induced quadriceps contraction increased irisin concentrations in serum and synovial fluid and enhanced irisin immunoreactivity in cartilage. Both interventions were associated with improved cartilage morphology, greater proteoglycan staining and type II collagen expression, and reduced MMP-9, MMP-13, ADAMTS-5, IL-1β, IL-6, IL-18, and TNF-α levels. Electrically induced muscle contraction elicited a greater increase in irisin and a more pronounced anti-inflammatory response than treadmill exercise, whereas indices of cartilage repair were broadly comparable between interventions [101]. Irisin levels were inversely correlated with inflammatory mediators; however, FNDC5/irisin signaling was not experimentally inhibited or genetically manipulated. Consequently, these associations do not establish irisin as the causal mediator of the exercise response.

Collectively, animal studies indicate that genetic or pharmacological enhancement of FNDC5/irisin signaling can attenuate selected structural, inflammatory, mitochondrial, and matrix-catabolic abnormalities in experimental OA [65]. Nevertheless, interpretation is constrained by model heterogeneity, early treatment initiation, limited dose–response and pharmacokinetic characterization, the use of potentially supraphysiological recombinant-protein concentrations, and the absence of independent clinical validation. Accordingly, these findings support the preclinical chondroprotective potential of irisin but do not establish disease-modifying efficacy in patients [102].

The main in vivo evidence supporting these mechanisms is summarized in Table 2.

### 3.2. Biomaterial-Based Strategies for Intra-Articular Irisin Delivery

In the absence of approved disease-modifying OA drugs, intra-articular administration offers a clinically accessible approach to achieving high local drug concentrations while limiting systemic exposure. However, its therapeutic efficacy is often constrained by rapid enzymatic degradation, synovial clearance, and limited tissue retention, particularly for proteins and peptides [103,104]. Biomaterial-based platforms—including hydrogels, nanoparticles, microspheres, liposomes, and composite carriers have therefore been developed to protect therapeutic agents, prolong their intra-articular residence, and provide spatially and temporally controlled release [105,106,107,108,109,110,111,112,113,114,115]. These properties may improve local bioavailability and prolong pharmacological activity while reducing injection frequency and systemic exposure [104,116].

Injectable hydrogels are particularly attractive for intra-articular delivery because of their biocompatibility, tunable physicochemical and mechanical properties, and capacity to reproduce selected features of the cartilage ECM [117]. Natural polymers investigated for OA-related applications include hyaluronic acid (HA), chitosan, alginate, chondroitin sulfate, gelatin, and silk fibroin [107,118,119,120,121,122,123]. Their composition and cross-linking density can be modified to regulate injectability, mechanical stability, degradation kinetics, drug-loading efficiency, and release profiles. In addition to functioning as delivery matrices, selected hydrogel formulations may provide a cell-compatible microenvironment that supports extracellular matrix deposition and tissue repair [104,116].

Stimulus-responsive, or “smart,” hydrogels provide an additional level of control by coupling gelation, ECM degradation, or cargo release to physicochemical features of the joint microenvironment. Thermoresponsive formulations can undergo in situ gelation after injection, whereas ROS-responsive systems can promote preferential material degradation or cargo release under oxidative conditions [118,123]. ROS-scavenging hydrogel platforms may additionally attenuate oxidative and ferroptotic stress within the OA joint [6]. These properties may be particularly relevant to irisin, whose intra-articular application could be limited by proteolytic degradation, conformational instability, and rapid clearance from the synovial cavity [103,104]. Encapsulation within an appropriately engineered hydrogel could preserve protein bioactivity and provide sustained local release. However, prolonged intra-articular retention does not necessarily ensure effective cartilage delivery, as tissue penetration is influenced by molecular size, charge, matrix-binding affinity, and carrier–cartilage interactions [103,104,124]. Hydrogel composition, cross-linking density, pore architecture, degradation kinetics, and irisin–matrix interactions would therefore require careful optimization to balance protein stabilization and controlled release with adequate cartilage exposure and preservation of receptor-binding competence.

The clinical use of HA viscosupplementation provides an established precedent for the intra-articular administration of polymer-based formulations. HA injections are used for the symptomatic management of OA, although their efficacy and duration of benefit vary across studies, formulations, and patient populations [124,125]. In addition to temporarily supplementing the viscoelastic properties of synovial fluid, HA may exert lubricating, anti-inflammatory, and chondroprotective effects, although the clinical relevance of these biological actions remains incompletely defined [124,125]. Moreover, the limited persistence of non-cross-linked HA formulations may restrict the duration of their effects and, depending on the product, require repeated administration [104,116].

Within this framework, biomaterial-assisted delivery may represent a promising strategy for translating the chondroprotective activity of irisin into a locally administered OA therapy. Encapsulation or immobilization within an injectable hydrogel could theoretically protect irisin from proteolytic degradation, prolong its residence within the synovial cavity, and provide sustained exposure to cartilage and synovial tissues. Importantly, however, the stability, pharmacokinetics, and tissue distribution of intra-articularly administered irisin have not yet been systematically characterized.

Preliminary evidence from other musculoskeletal applications supports the technical feasibility of biomaterial-mediated irisin delivery. Irisin has been incorporated into a silk fibroin/calcium silicate/sodium alginate composite scaffold for bone regeneration and, more recently, into a physically cross-linked silk fibroin hydrogel for sustained delivery in volumetric muscle-loss repair [126,127]. Nevertheless, no irisin-loaded biomaterial has yet been evaluated specifically for intra-articular administration or OA treatment. Future studies should therefore determine whether injectable or stimulus-responsive hydrogels can preserve irisin bioactivity, provide therapeutically relevant release kinetics, and enhance its retention and penetration within articular cartilage. Preclinical development should also include rigorous assessment of injectability, degradation behavior, mechanical compatibility, immunogenicity, local toxicity, and therapeutic efficacy in OA-specific models.

## 4. Discussion

The available evidence suggests that irisin represents a biologically relevant mediator of cartilage homeostasis and a potential molecular link between skeletal muscle activity and joint health. OA is increasingly recognized as a disease driven by complex interactions among inflammatory signaling, metabolic dysregulation, mitochondrial dysfunction, and ECM imbalance [2]. Within this multifactorial context, experimental evidence indicates that irisin modulates several pathways implicated in chondrocyte dysfunction and cartilage degeneration [82,85].

One of the most consistent observations emerging from experimental studies is the capacity of irisin to modulate the balance between anabolic and catabolic processes within the cartilage microenvironment. OA progression is characterized by excessive activation of catabolic enzymes, including MMPs and aggrecanases such as ADAMTS-5, which degrade key structural ECM components such as COL2 and ACAN [36]. In vitro studies demonstrate that irisin suppresses the expression of these enzymes while simultaneously enhancing anabolic markers, including COL2A1, ACAN, and SOX9. This dual regulatory effect suggests that this myokine does not simply inhibit cartilage degradation but actively promotes the restoration of ECM homeostasis.

In addition to its effects on ECM metabolism, irisin appears to exert potent anti-inflammatory actions in chondrocytes. Chronic low-grade inflammation is a central component of OA pathophysiology and is mediated, in part, by cytokines such as IL-1β and TNF-α [26,28,80]. These cytokines activate intracellular signaling cascades including NF-κB and MAPK pathways, leading to increased production of inflammatory mediators, oxidative stress, and apoptosis. Experimental evidence indicates that irisin suppresses NF-κB activation and reduces the nuclear translocation of p65, thereby limiting the transcription of pro-inflammatory cytokines and catabolic enzymes. The inhibition of MAPK signaling pathways, including p38 and JNK, further contributes to the attenuation of inflammatory responses. Through these mechanisms, irisin attenuates inflammatory and matrix-catabolic signaling in experimental chondrocyte models [74,76].

Another important aspect of irisin biology concerns its role in regulating mitochondrial homeostasis and cell stress responses. Increasing evidence indicates that mitochondrial dysfunction represents a critical driver of chondrocyte degeneration. Impaired mitochondrial respiration, excessive ROS production, and defective mitophagy contribute to oxidative damage and apoptosis in OA cartilage [39]. Irisin has been shown to counteract these processes by promoting mitochondrial biogenesis through PGC-1α–dependent signaling and by enhancing mitochondrial quality control via the PINK1/Parkin pathway [28]. These effects are accompanied by increased expression of mitochondrial regulatory proteins such as SIRT3 and TFAM, which support mitochondrial integrity and metabolic efficiency. These effects are associated with improved mitochondrial membrane potential and ATP production, reduced oxidative stress, and enhanced chondrocyte survival under inflammatory conditions [10,78,128].

The regulation of autophagy represents another mechanism through which the myokine may influence cartilage homeostasis. Autophagy is a critical cytoprotective process that maintains cellular homeostasis by removing damaged organelles and misfolded proteins. In OA cartilage, autophagic activity is often impaired, leading to the accumulation of cellular damage and increased susceptibility to apoptosis [79]. Experimental studies indicate that irisin restores autophagic flux by modulating the expression of key autophagy-related proteins, including LC3-II, Atg proteins, and p62 [82]. In addition, it appears to enhance mitophagy, a specialized form of autophagy that selectively removes dysfunctional mitochondria [56,129]. The coordinated activation of these quality control pathways may represent a central mechanism underlying the cytoprotective effects of irisin in chondrocytes.

Evidence from animal models further reinforces the biological relevance of irisin in OA pathophysiology. Reduced FNDC5/irisin expression has been observed in OA cartilage and is associated with increased oxidative DNA damage, impaired autophagy, and enhanced apoptosis [34,50,57]. Conversely, administration of recombinant irisin has been shown to attenuate cartilage degeneration in several experimental OA models. In these models, irisin treatment preserved cartilage structure, improved histological scores, and restored ECM composition. These effects were accompanied by reduced inflammatory signaling and enhanced cell survival, supporting the preclinical chondroprotective activity of irisin [74]. However, structural improvement in experimental OA models does not establish disease-modifying efficacy in patients.

The exercise-responsive nature of irisin provides a plausible biological link between skeletal muscle activity and joint adaptation. Skeletal muscle functions as a secretory organ capable of releasing myokines in response to contraction [6,7,8]. Exercise-induced FNDC5/irisin signaling may contribute to muscle–cartilage communication and modulate chondrocyte metabolism, inflammatory responses, and ECM homeostasis [73,76,101,102]. However, exercise induces concurrent biomechanical, neuromuscular, metabolic, inflammatory, and endocrine adaptations; therefore, its cartilage-related effects cannot be attributed specifically to irisin without direct manipulation of FNDC5/irisin signaling.

The interaction between mechanical loading and irisin-related responses is likely to be context-dependent. Physiological loading supports cartilage metabolism, whereas injurious or excessive loading promotes chondrocyte death and activates catabolic pathways associated with cartilage degeneration [32,33,80,130]. Mechanical stimulation has also been reported to modulate irisin-associated signaling and suppress chondrocyte pyroptosis in experimental OA. Accordingly, exercise should be considered a complex biological intervention whose effects depend on modality, intensity, duration, baseline joint status, and the prevailing mechanical environment. Studies incorporating receptor-specific or genetic manipulation will be required to distinguish irisin-dependent effects from the broader systemic response to physical activity.

### 4.1. Critical Appraisal and Translational Limitations of Current Experimental Models

The strength and translational relevance of the available evidence vary substantially across experimental models. Cell lines facilitate controlled mechanistic and dose–response analyses but may not adequately reproduce the phenotype of primary human articular chondrocytes. Primary OA chondrocytes provide direct human disease-derived evidence; however, their interpretation is constrained by donor heterogeneity, frequent derivation from end-stage disease, culture-induced dedifferentiation, and typically short experimental exposures. Three-dimensional culture systems better preserve chondrogenic differentiation and ECM deposition than conventional monolayer cultures but cannot recapitulate the synovial, immune, vascular, and biomechanical complexity of the whole joint.

The study conducted by members of the present author group used human OA chondrocytes in a three-dimensional pellet culture system and reported anabolic, anti-inflammatory, and anti-catabolic responses following exposure to recombinant irisin [74]. The use of human OA-derived cells and a three-dimensional model represents a methodological strength. Nevertheless, interpretation is limited by the in vitro design, the number and heterogeneity of donor-derived cell preparations, the relatively short experimental duration, the use of exogenous recombinant irisin, the absence of the complete joint microenvironment, and the lack of independent replication. The study did not establish whether the experimental exposure reflected physiologically achievable concentrations in human joint tissues and did not investigate direct ligand binding, receptor identity, or receptor-dependent target engagement. These findings should therefore be considered preliminary mechanistic evidence and do not establish therapeutic efficacy, disease-modifying activity, or clinical translatability.

Similar caution is warranted when interpreting the remaining in vitro evidence. Studies using transformed cell lines provide experimentally reproducible systems for investigating molecular pathways and dose-dependent responses, but their biological relevance to native articular cartilage is limited [84]. Experiments involving primary chondrocytes [76,82,98] or human MSCs [75] offer greater physiological relevance; nevertheless, differences in cell source, donor characteristics, disease stage, culture conditions, recombinant irisin preparations, concentrations, and exposure durations substantially limit direct comparisons across studies. Moreover, the observed modulation of pathways such as ERK1/2, NF-κB, MAPK, PI3K/Akt, and Wnt/β-catenin demonstrates cellular responsiveness to irisin but does not, by itself, identify the receptor responsible for these effects. Overall, the available in vitro evidence supports the biological plausibility of an anabolic and chondroprotective action of irisin, but its reproducibility and physiological relevance require confirmation in independent laboratories using standardized experimental conditions and receptor-specific approaches.

Rodent OA models permit tissue-level evaluation of cartilage structure, synovial responses, subchondral bone, and whole-joint interactions [65,82,100,101]. However, species-specific differences in cartilage anatomy, joint biomechanics, disease induction, metabolism, and pharmacokinetics limit direct extrapolation to human OA. In addition, early intervention in many experimental models more closely reflects prevention or attenuation of developing OA-like changes than treatment of established human disease.

Exercise-based models introduce further interpretative complexity because their outcomes reflect concurrent mechanical, metabolic, inflammatory, and endocrine adaptations and cannot be attributed exclusively to irisin. Studies that report associations between exercise-induced changes in irisin and cartilage outcomes provide indirect evidence of muscle–cartilage crosstalk but cannot establish irisin as the causal mediator unless FNDC5/irisin signaling is specifically inhibited or genetically manipulated [101].

Experimental studies also differ substantially in the source, purity, formulation, and biochemical characterization of recombinant irisin, as well as in the concentrations tested and duration of exposure. The use of incompletely characterized preparations or potentially supraphysiological exposure levels limits cross-study comparability and complicates the assessment of physiological and pharmacological relevance. Accordingly, molecular, cellular, histological, or structural improvements observed in experimental models should be interpreted as evidence of biological activity and preclinical chondroprotective potential rather than as proof of clinical efficacy or disease modification in humans. The principal strengths, limitations, and translational implications of the available evidence are summarized in Table 3.

### 4.2. Analytical Limitations and Biomarker Readiness

The clinical interpretation of circulating and synovial irisin is constrained by substantial analytical and pre-analytical heterogeneity. Reported concentrations vary according to assay platform, antibody specificity, calibration standard, sample matrix, specimen handling, and the molecular forms of FNDC5/irisin detected [131,132,133]. Commercial immunoassays may differ in their ability to distinguish mature irisin from precursor-derived or cross-reactive immunoreactive products. Mass-spectrometry-based methods provide greater molecular specificity but remain less accessible and are not yet standardized for routine clinical application.

Pre-analytical sources of variability include the timing of sample collection relative to exercise, fasting status, circadian variation, collection-tube characteristics, time to processing, centrifugation conditions, storage temperature, and the number of freeze–thaw cycles. Biological variability may additionally reflect age, sex, recent physical activity, body composition, metabolic status, and renal function [134,135]. However, much of the evidence supporting these associations derives from heterogeneous, non-OA populations, and the direction and magnitude of the reported effects are not consistent across studies. Medication use and comorbidities should therefore be recorded as potential confounding variables, although their specific effects on irisin concentrations remain insufficiently characterized.

Synovial-fluid measurements are subject to additional matrix-specific challenges related to high viscosity, sampling procedures, aspirated volume, dilution introduced by lavage, cellular and debris content, local inflammatory activity, and disease stage. These factors may influence assay recovery, linearity, precision, and interstudy comparability, thereby requiring standardized collection, pretreatment, dilution-correction, and storage procedures [136,137].

Consequently, neither circulating nor synovial irisin currently fulfills the requirements for use as a validated diagnostic, prognostic, predictive, or treatment-response biomarker in OA. The available human evidence is predominantly cross-sectional, frequently involves small or heterogeneous cohorts, and lacks established reference intervals, clinically meaningful thresholds, independent validation, and prospective assessment of incremental value over existing clinical, imaging, and biochemical measures. Before biomarker applicability can be established, future studies will require validated reference materials, harmonized assay procedures, cross-platform comparisons, prespecified pre-analytical protocols, matrix-specific analytical validation, and adequately powered prospective cohorts. These considerations support the interpretation of serum and synovial irisin as exploratory research measures rather than clinically applicable OA biomarkers.

## 5. Conclusions

Irisin has emerged as a key mediator at the intersection between skeletal muscle activity and cartilage biology. Experimental evidence indicates that this exercise-induced myokine exerts chondroprotective effects by suppressing inflammatory and catabolic signaling, promoting ECM synthesis, and preserving mitochondrial function and autophagic balance in chondrocytes. Although current data are largely derived from experimental models, the consistent protective effects observed across in vitro and in vivo studies support the hypothesis that irisin plays a relevant role in the preservation of joint homeostasis. These findings also provide a mechanistic framework linking physical activity to cartilage protection, suggesting that irisin may contribute to the beneficial effects of exercise in osteoarthritis. Further research is needed to clarify the molecular mechanisms of irisin signaling in cartilage, identify its receptor systems, and determine whether circulating or synovial irisin levels may serve as reliable biomarkers of disease activity. In parallel, the development of pharmacological or biomaterial-based strategies capable of harnessing irisin signaling may open new perspectives for disease-modifying therapies in osteoarthritis.

## Figures and Tables

**Table 1:** Summary of in vitro studies evaluating the effects of irisin on chondrocytes and related cell models.

Study	Model	Experimental Conditions	Main Findings	Key Mechanisms
Vadalà et al. [74]	Human OA chondrocytes (3D pellet)	Irisin (dose–response study: 25, 50, 75, and 100 ng/mL; 25 ng/mL used for subsequent analyses)	↑ GAG synthesis, ↑ cell content, ↑ COL2A1, ↓ COL10A1, ↓ IL-1β, IL-6, ↓ MMP-1, MMP-13, ↑ TIMP-1, TIMP-3	↓ NF-κB, ↓ MAPK (p38, JNK), ↓ Akt
Li et al. [84]	SW1353 chondrosarcoma cells	IL-1β (dose–response study: 0, 5, 10, 20, and 50 ng/mL; 10 ng/mL used for subsequent analyses) ± irisin (dose–response study: 0, 10, 20, 50, and 100 mM; 20 mM used for subsequent analyses)	↑ COL2A1, ↓ MMP-13, ↓ Wnt/β-catenin, ↓ NF-κB, p65	↓ Wnt/β, NF-κB
Posa et al. [98]	Human chondrocytes (3D culture)	Irisin (100 ng/mL)	↑ SOX9, ACAN, COL2A1, ↑ ECM deposition, no hypertrophy	↑ ERK
Chen et al. [75]	hMSCs	Irisin (100 ng/mL)	↑ chondrogenesis, ↑ COL2A1, ACAN, SOX9	miR-125b-5p → ↓ SIPA1L2 → ↑ Rap1 and PI3K/Akt/mTOR
Jia et al. [76]	Primary OA chondrocytes	IL-1β (10 ng/mL) ± irisin (5 and 10 ng/mL)	↑ COL2A1, ↓ MMP-13, ↓ ADAMTS-5, ↓ pyroptosis	↓ PI3K/Akt/NF-κB, ↓ NLRP3/caspase-1
Wang et al. [82]	Primary mouse OA chondrocytes	IL-1β (5 ng/mL) ± irisin (1, 5, and 10 ng/mL)	↑ GAG, ↑ COL2A1, ↓ ROS, ↑ mitochondrial function	↑ SIRT3, ↑ autophagy, ↑ mitochondrial dynamics

Arrows indicate the regulation status of the indicated pathways: ↑, upregulation; ↓, downregulation; and →, stability (no change). Abbreviations: ACAN: aggrecan; ADAMTS: a disintegrin and metalloproteinase with thrombospondin motifs; COL10A1: type X collagen alpha 1 chain; COL2A1: type II collagen alpha 1 chain; ECM: extracellular matrix; ERK: extracellular signal-regulated kinase; GAG: glycosaminoglycan; hMSCs: human mesenchymal stromal cells; IL: interleukin; JNK: c-Jun N-terminal kinase; MAPK: mitogen-activated protein kinase; MMP: matrix metalloproteinase; NF-κB: nuclear factor kappa B; NLRP3: NOD-, LRR- and pyrin domain-containing protein 3; OA: osteoarthritic; PI3K: phosphatidylinositol 3-kinase; Rap1: RAS-associated protein 1; ROS: reactive oxygen species; SIPA1L2: signal induced proliferation associated 1 Like 2; SIRT3: sirtuin 3; SOX: SRY-box transcription factor; TIMP: tissue inhibitor of metalloproteinase.

**Table 2:** Summary of in vivo studies evaluating the effects of irisin on animal models of OA.

Study	Model	Experimental Conditions	Main Findings	Key Mechanisms
Wang et al. [82]	Mouse (DMM)	IA recombinant irisin (20 mg/mL in 10 μL)	↓ Cartilage damage, ↓ synovitis, improved gait; ↓ irisin in OA tissue	↑ Autophagy (LC3-II), ↓ apoptosis, ↑ mitochondrial function (SIRT3, PGC-1α)
Li et al. [65]	Mouse (irisin KO/KI; ACLT)	Irisin genetic deletion/overexpression; IA recombinant irisin (1 μg/μL in 5 μL every 3 days for 8 weeks)	KO: ↑ OA severity; treatment: ↓ cartilage degeneration	↑ ECM homeostasis (↑ COL2A1, ACAN), ↓ hypertrophy (COL10A1)
Açan et al. [100]	Rat (FTCD)	IA recombinant irisin (10 μg/mL in 100 μL) ± bevacizumab (12.5 mg/0.5 mL)	↑ Cartilage repair, ↓ defect size, hyaline-like tissue formation	Pro-regenerative effect; synergy with anti-angiogenic signaling
Duan et al. [101]	Rat (MIA-induced OA + exercise)	Exercise-induced irisin: treadmill exercise (19.3 m/min, 60 min/day) vs. electroacupuncture-induced passive muscle contraction (1–3 mA, 1 Hz, 15 min/session)	↑ Cartilage integrity, ↓ inflammation, ↑ COL2A1	↓ Pro-inflammatory cytokines, ↓ MMPs, ↑ ECM synthesis

Arrows indicate the regulation status of the indicated pathways: ↑, upregulation; ↓, downregulation. Abbreviations: ACAN: aggrecan; ACLT: anterior cruciate ligament transection; COL10A1: type X collagen alpha 1 chain; COL2A1: type II collagen alpha 1 chain; DMM: destabilization of the medial meniscus; ECM: extracellular matrix; FTCD: full-thickness cartilage defect; IA: intraarticular; KI: knock-in; KO: knockout; LC3-II: light chain 3 beta II; MIA: monoiodoacetate; MMP: matrix metalloproteinase; OA: osteoarthritic; PGC-1α: peroxisome proliferator-activated receptor gamma coactivator-1 alpha; SIRT3: sirtuin3.

**Table 3:** Critical appraisal of the evidence supporting the potential chondroprotective effects of irisin.

Evidence Model	Principal Strengths	Main Limitations	Translational Interpretation
Cell lines	Controlled mechanistic and dose–response analyses; experimental reproducibility	Immortalized or transformed phenotype; limited physiological fidelity	Hypothesis-generating mechanistic evidence
Primary OA chondrocytes	Direct assessment of human disease-derived cells	Donor heterogeneity; frequent derivation from end-stage OA; culture-induced dedifferentiation; short exposure periods	Direct human-cell evidence with limited capacity to predict clinical efficacy
Three-dimensional cultures	Improved preservation of the chondrogenic phenotype and ECM deposition	Absence of synovial, immune, vascular, and physiological loading interactions	Intermediate mechanistic and tissue-level relevance
Rodent OA models	Assessment of whole-joint structural responses and tissue interactions	Species-specific anatomy and biomechanics; model-dependent disease induction; differences in dosing, metabolism, and pharmacokinetics	Preclinical proof of concept; insufficient to establish human disease modification
Exercise-based models	Evaluation of endogenous irisin within a physiologically relevant intervention	Multiple concurrent mechanical, metabolic, inflammatory, and endocrine effects; limited causal attribution to irisin	Indirect support for exercise-mediated muscle–cartilage crosstalk

Abbreviations: ECM, extracellular matrix; OA, osteoarthritis.

## Data Availability

No new data were created or analyzed in this study. Data sharing is not applicable to this article.
